# 肺癌患者发病情况和经济负担的分析

**DOI:** 10.3779/j.issn.1009-3419.2022.101.09

**Published:** 2022-03-20

**Authors:** 国 田, 莉 边, 小莉 徐, 书梅 李

**Affiliations:** 1 050000 石家庄，河北医科大学第四医院（河北省肿瘤医院）病案室 Department of Medical Record, The Fourth Hospital of Hebei Medical University (Hebei Tumor Hospital), Shijiazhuang 050000, China; 2 250017 济南，山东省肿瘤防治研究院（山东省肿瘤医院），山东第一医科大学（山东省医学科学院） Shandong Cancer Hospital and Institute, Shandong First Medical University and Shandong Academy of Medical Sciences, Jinan 250017, China

**Keywords:** 肺肿瘤, 性别, 年龄, 住院天数, 住院费用, Lung neoplasms, Gender, Age, Days of hospitalization, Hospital costs

## Abstract

**背景与目的:**

肺癌在我国具有较高的发病率和致死率，并造成了较大的经济负担，本研究旨在通过分析河北省肿瘤医院肺癌患者出院病历首页的相关信息，研究肺癌的发病情况和经济负担，为肺癌的防治研究提供科学依据。

**方法:**

基于病案管理系统检索出2012年1月-2019年12月河北省肿瘤医院肺癌患者的出院人次、新发人数、手术人数、年龄、性别、住院天数和住院费用等信息，统计描述肺癌患者发病趋势、性别和年龄分布以及疾病的经济负担。

**结果:**

肺癌新发人数逐年增加，2012年肺癌新发2, 235人，2019年为5, 012人。发病人数男性始终大于女性，但男女性别比逐年降低，由2012年的2.25降至2019年的1.56。肺癌新发人数中手术治疗的比例逐年增加，由2012年的28.14%增长到2019年的44.83%。除2012年外，2013年-2019年女性患者的手术比例均高于男性，并且逐年增加，2013年新发肺癌男女患者的手术比例分别为23.52%和28.07%，2019年分别为36.14%和58.37%。肺癌患者的中位发病年龄逐年增长，2012年新发肺癌患者的中位年龄为61岁，2019年为63岁。全部肺癌患者的中位发病年龄，男性均大于女性。肺癌新发患者和手术患者的人数都显示出随着年龄的增长而增加的趋势，且均在60岁-69岁的年龄组段达到最大值，随着年龄的增长，患者人数逐渐减少，所有年龄组段的发病人数也逐年增加。所有出院人次的肺癌患者和手术患者的中位住院天数逐年减少，分别由2012年的10 d和19 d降低至2019年的8 d和17 d，而中位住院费用逐年升高，分别由2012年的10, 611.46元和38, 750.13元增长到2019年的17, 187.15元和84, 030.16元。

**结论:**

肺癌仍是危害我国居民健康的主要癌种之一，发病人数逐年增加，性别和年龄分布具有一定的特征，应制定有效的防控措施，加强医疗改革，减少发病人数和经济负担。

## Abstract (en)

**Background and objective:**

The previous study has indicated that lung cancer has a high incidence and mortality in China, and has caused a large economic burden. The purpose of this study was to analyze the incidence and economic burden of lung cancer by analyzing the information on the home page of discharge history of lung cancer patients in Hebei Tumor Hospital, and to provide scientific basis for the prevention and treatment of lung cancer.

**Methods:**

The information of all of the discharges, new cases, surgical patients, age, gender, length of stay and hospitalization cost of lung cancer patients in Hebei Tumor Hospital from January 2012 to December 2019 were retrieved based on the medical record management system, and the incidence trend, gender and age distribution as well as the economic burden of the disease were statistically described.

**Results:**

The number of new cases of lung cancer increased year by year, from 2, 235 cases in 2012 to 5, 012 cases in 2019. The number of males always outnumbered females, but the gender ratio decreased year by year, from 2.25 in 2012 to 1.56 in 2019. Among new cases of lung cancer, the proportion of surgical treatment increased year by year, from 28.14% in 2012 to 44.83% in 2019. Except for 2012, the proportion of surgical operations in female patients was higher than that in male patients from 2013 to 2019. The proportion of surgical operations in male and female patients was 23.52% and 28.07% in 2013, and 36.14% and 58.37% in 2019, respectively. The median age at the onset of lung cancer has increased year by year, from 61 years old in 2012 to 63 years old in 2019. The median age of onset in all lung cancer patients was higher in males than in females. The number of new lung cancer patients and surgical patients both showed an increasing trend with the increase of age, and both reached the maximum value in the age group of 60-69 years old. With the increase of age, the number of patients gradually decreased. The median length of hospital stay for all discharged lung cancer patients or surgical patients decreased year by year, from 10 d and 19 d in 2012 to 8 d and 17 d in 2019, respectively, while the median hospitalization cost increased year by year. It increased from 10, 611.46 yuan and 38, 750.13 yuan in 2012 to 17, 187.15 yuan and 84, 030.16 yuan in 2019, respectively.

**Conclusion:**

Lung cancer is still one of the main cancers endangering the health of Chinese residents. The incidence of lung cancer is increasing year by year, and the distribution of gender and age has certain characteristics. In order to reduce the number of cases and the economic burden, effective prevention and control measures should be formulated and medical reform should be strengthened.

肺癌严重危害着人类健康，是全球高发病率和死亡率的主要恶性肿瘤^[1]^。在我国，肺癌的粗发病率和粗死亡率均居所有恶性肿瘤的首位，分别为54.66/10万和45.60/10万^[2]^。其中，河北省肺癌的发病率和死亡率在过去40年显著增长，尤其是死亡增长率为189.15%^[3]^。肺癌的发病率和死亡率居高不下，对人民的生命健康造成了威胁，并且治疗需要花费高额费用，造成了较大的经济负担，因此积极开展肺癌的防治工作尤其重要。河北省肿瘤医院每年收治大量的肺癌患者，出院病历详细地记录了所有患者的基本信息和住院信息。本研究旨在通过收集患者出院病历首页的相关信息描述肺癌患者的发病趋势、分布特征和住院费用等情况，并分析其原因，为肺癌患者的防治研究提供科学依据。

## 1 资料与方法

### 1.1 资料来源

采集2012年1月-2019年12月河北省肿瘤医院所有肺癌出院患者的病历首页信息，包括出院人次、新发人数、手术人数、年龄、性别、住院天数和住院费用。

### 1.2 数据检索标准

基于病案管理系统进行检索，按照国际疾病分类（international classification of diseases, ICD）中肿瘤编码的原则结合该院住院患者只使用唯一住院号的特点，新发肺癌患者信息的检索标准为病历首页中主要诊断的ICD-10编码在C34区间，住院次数为最小，同时删除少数因复发或者再次手术等原因而具有相同编码且在往年住院的患者信息；所有肺癌患者信息的检索是以病历首页的主要诊断进行关键字“肺”和“癌”或“瘤”等名称的模糊查询，然后再删除不符合条件的患者信息；手术患者的检索标准需要同时满足主要诊断的ICD-10编码在C34区间且所有手术ICD-9-CM-3的编码在32.0-32.9区间。

### 1.3 统计学方法

所有的病历首页信息导出后，采用Excel 2016对肺癌患者人数、性别比、年龄组段的分布进行整理，通过SPSS 25.0软件对年龄、住院天数和住院费用等指标的差异采用非参数检验进行统计描述和分析，以*P* < 0.05为差异有统计学意义。

## 2 结果

### 2.1 肺癌新发人数、手术人数及性别分布

肺癌新发人数逐年增加，2012年发病人数为2, 235人，2019年为5, 012人。发病人数男性始终大于女性，但男女性别比例逐年降低，由2012年的2.25降至2019年的1.56。2012年-2018年男性患者手术人数均大于女性，性别比同样逐年降低，直到2019年的男性患者的手术人数1, 103人少于女性1, 144人。肺癌新发人数中手术治疗的比例逐年增加，由2012年的28.14%增长到2019年的44.83%。除2012年外，2013年-2019年女性患者的手术比例均高于男性，并逐年增加，2013年新发肺癌男女患者的手术比例分别为23.52%和28.07%，2019年则分别为36.14%和58.37%（表 1）。

**表 1:** 2012年-2019年肺癌新发人数、手术人数、性别比及手术比例的分布 Distribution of new lung cancer cases, number of operations, gender ratio and surginal proportion from 2012 to 2019

Year	New cases (*n*)					Surgical cases (*n*)					Surgical proportion (%)		
	Total	Male	Female	Gender ratio		Total	Male	Female	Gender ratio		Total	Male	Female
2012	2, 235	1, 548	687	2.25		629	443	186	2.38		28.14	28.62	27.07
2013	2, 613	1, 790	823	2.17		652	421	231	1.82		24.95	23.52	28.07
2014	2, 871	1, 890	981	1.93		826	526	300	1.75		28.77	27.83	30.58
2015	3, 159	2, 119	1, 040	2.04		839	493	346	1.42		26.56	23.27	33.27
2016	3, 366	2, 194	1, 172	1.87		1, 013	592	421	1.41		30.1	26.98	35.92
2017	3, 961	2, 547	1, 414	1.80		1, 345	731	614	1.19		33.96	28.7	43.42
2018	4, 288	2, 733	1, 555	1.76		1, 662	876	786	1.11		38.76	32.05	50.55
2019	5, 012	3, 052	1, 960	1.56		2, 247	1, 103	1, 144	0.96		44.83	36.14	58.37

### 2.2 不同性别的肺癌新发及手术患者的年龄分布

肺癌新发患者的中位年龄逐年增加，2012年的新发肺癌患者的中位年龄为61岁，2019年为63岁，无论是男性还是女性，中位发病年龄均呈现出上升趋势，且男性大于女性（*P* < 0.05）。手术患者中位发病年龄由2012年的60岁增长到2019年的62岁，同样男性均大于女性（*P* < 0.05）（表 2）。

**表 2:** 2012年-2019年男女肺癌新发患者及手术患者的年龄分布 Age distribution of new lung cancer cases and surgical cases in male and female from 2012 to 2019

Year	Age of new cases (yr)					Age of surgical cases (yr)			
	Total	Male	Female	*P*		Total	Male	Female	*P*
2012	61	61	59	< 0.001		60	61	59	0.049
2013	61	62	60	< 0.001		59	60	58	0.009
2014	61	62	60	< 0.001		61	62	60	0.003
2015	62	62	61	< 0.001		60	62	59	0.001
2016	62	63	61	< 0.001		61	61	60	0.023
2017	63	63	62	< 0.001		61	62	60	< 0.001
2018	63	64	62	< 0.001		62	63	61	0.001
2019	63	64	62	< 0.001		62	63	60	< 0.001

### 2.3 不同年份的肺癌新发及手术患者的年龄组分布

本研究共划分7个年龄组，最小为29岁以下，最大为80岁以上，其余年龄组段的间距为10岁。肺癌新发患者和手术患者的人数均显示出了随着年龄的增长而增加的趋势，均在60岁-69岁的年龄组达到最大，70岁后人数逐渐减少，大多年龄组段的新发人数和手术人数逐年增加（表 3）。

**表 3:** 2012年-2019年肺癌新发患者和手术患者的年龄组段分布 Age group distribution of new lung cancer cases and surgical cases from 2012 to 2019

Year	0-29 (yr)			30-39 (yr)			40-49 (yr)			50-59 (yr)			60-69 (yr)			70-79 (yr)			≥80 (yr)	
	New cases	Surgical cases		New cases	Surgical cases		New cases	Surgical cases		New cases	Surgical cases		New cases	Surgical cases		New cases	Surgical cases		New cases	Surgical cases
2012	11	2		46	10		272	67		676	217		809	258		378	74		43	1
2013	13	6		44	7		266	71		818	248		992	244		426	76		54	0
2014	15	5		54	8		280	80		839	255		1, 189	372		429	102		65	4
2015	12	2		50	10		255	75		913	289		1, 336	377		514	84		79	2
2016	14	5		62	15		295	96		899	311		1, 436	439		579	139		81	8
2017	10	2		81	33		325	137		1, 007	383		1, 743	583		698	199		97	8
2018	17	6		81	40		308	126		1, 047	458		1, 935	774		819	244		81	14
2019	6	6		100	57		348	176		1, 261	689		2, 254	993		954	313		89	13

### 2.4 不同年份全部出院人次肺癌患者和手术患者的住院天数和费用的分布

无论是所有出院人次的肺癌患者还是手术患者的中位住院天数均逐年减少，分别由2012年的10 d和19 d降低至2019年的8 d和17 d。所有出院人次的肺癌患者和手术患者的中位住院费用逐年升高，前者的中位住院费用由2012年的10, 611.46元增长到2019年的17, 187.15元。后者的中位住院费用由38, 750.13元增长至84, 030.16元（表 4，表 5）。

**表 4:** 2012年-2019年所有出院人次肺癌患者和手术患者的中位住院天数 Median length of hospital stay of all lung cancer cases and surgical cases from 2012 to 2019

Year	All lung cancer cases (d)					Surgical lung cancer cases (d)			
	Total	Male	Female	*P*		Total	Male	Female	*P*
2012	10	10	9	0.547		19	20	18	0.003
2013	9	9	8	< 0.001		18	19	18	0.002
2014	9	9	9	0.042		18	18	17	< 0.001
2015	9	9	9	< 0.001		17	18	17	< 0.001
2016	9	9	9	< 0.001		17	18	16	< 0.001
2017	9	9	9	0.001		17	17	16	< 0.001
2018	9	9	9	0.001		17	18	16	< 0.001
2019	8	9	8	< 0.001		17	17	16	< 0.001

**表 5:** 2012年-2019年所有出院人次肺癌患者和手术患者的中位住院费用 Median hospitalization cost of discharges of all lung cancer cases and surgical cases from 2012 to 2019

Year	All lung cancer cases (yuan)					Surgical lung cancer cases (yuan)			
	Total	Male	Female	*P*		Total	Male	Female	*P*
2012	10, 611.46	10, 872.47	10, 046.42	< 0.001		38, 750.13	39, 276.02	36, 456.98	< 0.001
2013	10, 940.03	11, 364.10	10, 337.56	< 0.001		42, 321.58	42, 821.42	41, 486.33	0.001
2014	11, 857.79	11, 936.70	11, 624.00	0.010		47, 060.98	47, 421.69	46, 869.51	0.131
2015	12, 657.21	13, 084.15	11, 990.22	< 0.001		51, 870.54	53, 354.02	49, 406.27	< 0.001
2016	13, 070.10	13, 169.32	12, 831.39	0.005		56, 755.89	57, 755.46	55, 477.91	0.025
2017	15, 423.00	15, 390.26	15, 491.95	0.075		66, 255.08	68, 290.91	63, 487.26	< 0.001
2018	15, 953.39	15, 754.84	16, 461.88	< 0.001		71, 790.32	72, 429.65	70, 928.13	< 0.001
2019	17, 187.15	17, 027.86	17, 512.75	< 0.001		84, 030.16	85, 275.21	83, 167.09	< 0.001

## 3 讨论

本研究结果可见无论在男性还是女性患者中，肺癌发病人数逐年增加。在河北省肿瘤医院所在的石家庄市，肺癌的发病率在男性所有肿瘤中居第一位，在女性中仅次于乳腺癌居第二位，死亡率在男性和女性中均居第一位^[4]^。肺癌发病人数的增加与诸多因素有关，包括吸烟、大气污染和车辆排放的尾气等^[5]^，并且吸烟和空气污染是影响肺癌患者死亡和伤残调整生命年（disability-adjusted life year, DALY）的前两位主要原因^[6]^。吸烟已被证实是我国华北地区肺癌的重要危险因素^[7]^，已有研究^[8]^证实加强无烟条例的政策可以显著降低肺癌的发病率，通过改进和扩大烟草控制政策的实施可以预防未来20年欧洲范围内16, 500万肺癌病例^[9]^。由此可见，加强戒烟控烟行动势在必行。空气污染物中的PM1、PM2.5和臭氧等都与肺癌的发病率呈正相关^[10, 11]^，因此加强空气污染的治理措施对于肺癌的预防至关重要，相关研究^[12]^证实如果北京的空气质量在2014年-2016年达到国家长期PM2.5（35 μg/m^3^）标准，可预防10, 003例肺癌病例和438万元人民币的直接住院医疗费用，显著降低肺癌负担和节省医疗开支。

手术人数和手术比例的增加，主要与近年来抗癌知识的宣传和居民自我防护意识的增强有关，坚持定期体检能够早期发现病变。研究^[13]^发现，通过早诊早治筛选肺癌高危人群可有效提高阳性结果的检出率。而男女发病比例的降低，则提示越来越多的女性罹患肺癌，这与全球和国内女性肺癌上升的趋势相一致^[14-16]^。由于烟草控制措施并未针对家庭环境，因此女性接触被动吸烟的时间可能会持续更长^[17]^，其次厨房油烟等因素也对女性健康构成了巨大威胁^[18]^。研究^[19]^发现烹饪时间越长，尤其是每日烹饪时间 > 2 h，肺癌发病风险越大。不同于男性患者，女性肺癌有其独特的分子表达谱、组织病理学及激素代谢特征^[20-22]^，这些或许也是女性肺癌发病增多的原因。

无论是男性还是女性肺癌患者的发病年龄逐年增加，这可能与我国的老龄化有关。中国已于1999年进入老龄化社会，2001年-2020年的20年时间，中国平均每年新增596万老年人口，年均增长率达到3.3%，可见肺癌的发病率及发病年龄将持续增加^[23]^。已有研究^[24]^预测2035年将有1, 400万老年人新发癌症病例，占全球癌症发病率的60%左右，其中我国的发病率相对增加幅度较大，为155%。而手术患者年龄的增加，则体现了医疗手术水平的提高，克服了部分老年人因其年龄因素及自身并发症等原因不能手术的困难，加强老年患者围手术期处理、结合肿瘤外科的基本原则并采取合理的手术方式，绝大多数老年肺癌患者选择手术治疗是安全的^[25]^。男性发病年龄大于女性，影响因素是多方面的，或许是近年来提倡的戒烟限烟政策使相当部分的男性远离了烟草对肺癌的影响。女性或许过多地接触厨房油烟污染等其他危险因素，而厨房油烟大是女性肺癌除烟草外最大的发病风险因素^[26]^。这可能是导致男性肺癌发病年龄相对推后、女性发病年龄提前的部分原因。肺癌发病和死亡呈现出逐年上升的趋势^[27]^，尤其是在老年人群中。本研究中各个年龄组段的确诊人数普遍增加，包括许多年轻患者，很大原因得益于癌症的筛查，使其早发现、早诊断、早治疗，避免直到晚期才发现，由此可见，癌症筛查对于早期发现肺癌有着重要意义。研究^[28]^表明低剂量螺旋计算机断层扫描（computed tomography, CT）作为筛查的主要手段可以有效地检测出肺癌和肺结节阳性患者。肺癌患者的确诊人数在60岁-69岁的年龄段达到了最大值，原因之一是致癌因素的累积，如吸烟对于肺癌发生的剂量效应关系，吸烟越多，时间越长，致病危险越大。除此之外，老年人肺癌的高发还可能与自身生理因素进行性衰退、免疫功能下降及组织细胞对致癌物质易感性增高等有关^[29]^。

我国肺癌住院人次的年均增长率高达10%，其指数增长的势头依然处于加速阶段^[30]^。因肺癌的治疗是一个连续的过程，且大多数需要多次住院，所以住院费用成了肺癌患者的主要经济负担。本研究中手术患者的中位住院天数和费用均大于所有患者，是因为手术的耗材多、技术难度大、风险大、康复时间长等。随着年份的推移，患者的住院天数减少，这与现在医护水平的提高、治疗的规范化有关，提高了病床利用率、周转率，从而减少了住院天数。住院费用逐年增加，可能与新药物的研发、手术的设备更新有关。住院费用受病情和并发症影响较大。近年来，肺癌治疗的化疗药物、免疫药物、靶向药物等更新快，效果好，价格较高，能够应用于晚期肺癌患者，但是会直接导致经济负担的增加^[31]^。患者住院期间治疗因手术或者放化疗等原因导致的并发症也会增加住院费用^[32, 33]^。不同手术方式的选择同样会造成住院费用的差异。相关研究^[34]^表明胸腔镜肺叶切除手术的住院费用要高于开胸手术，电视胸腔镜手术（video-assisted thoracic surgery, VATS）的住院时间不仅少于开胸手术，而且能够降低术后疼痛和提高生活质量^[35]^，所以手术方式优先选择胸腔镜。近年来大多数肺癌患者都是通过癌症筛查或者定期体检发现的，肺癌组织相对较小，更容易选择创伤小的胸腔镜手术，所以手术患者的住院费用逐年增高。据报道^[36]^肺癌治疗的总费用极高，且国内肺癌患者的治疗效率和医疗保险受益公平性问题比较严重，应扩大医疗保险覆盖面。肺癌患者住院费用增长迅速，受政策影响较大。故需加强医药卫生体制改革，减轻患者及社会的经济负担^[37]^。

总之，肺癌仍然是危害我国居民健康的主要恶性肿瘤，其发病率一直居高不下，尤其是女性患者发病人数增加显著。肺癌患者仍以老年人为主，但目前年轻化趋势明显，并且造成了很大的经济负担。因此应提倡健康的生活方式，如锻炼身体，保持良好心态、戒烟限酒、远离空气污染，积极摄入水果、蔬菜，定期体检等。医疗部门也应追求更加规范化的诊断和治疗，提高早诊率，使其病情及时被发现，不被耽误；提高病床周转率，便于更快地服务于每一个患者，有效减少患者的住院时间；加强医疗改革，扩大医保覆盖面，从而减少患者经济负担。
